# Research on the Purchase Intention of Social Commerce Consumers in Video Streams: Dual Pathways of Affection and Rationality

**DOI:** 10.3390/bs14090738

**Published:** 2024-08-24

**Authors:** Minwei Deng, Yitong Yang, Baiqing Sun

**Affiliations:** 1 School of Management, Harbin Institute of Technology, Harbin 150001, China; 19b910026@stu.hit.edu.cn; 2 School of Applied Engineering, Henan University of Science and Technology, Sanmenxia 472000, China; yangyitong0402@163.com

**Keywords:** social commerce, live streaming, consumer behavior, purchase intention, field theory

## Abstract

Social commerce blurs the boundary between online social interaction and online shopping. The emergence of video streams introduces novel marketing modalities to social commerce. However, there is a paucity of comprehensive studies investigating the impact of emerging marketing techniques such as short videos and live streaming on consumer purchase intention. This study employs Bourdieu’s conceptual framework to construct a Field Theory-based model, investigating the impact of atmospheric and capital characteristics of social commerce platforms on consumer purchase intention through affective and rational pathways, respectively. A survey involving 515 Chinese social commerce consumers demonstrates that atmospheric characteristics (emotion and social presence) and capital characteristics (information quality and quantity) in video streams enhance similarity and power. Both similarity and power are associated with an increase in consumer purchase intention. This study validates the dual-path influence of social commerce characteristics and discusses theoretical and managerial implications.

## 1. Introduction

We Are Social has published the “Global Digital Report 2023”, indicating that individuals now spend over 2.5 h daily on social media platforms, an increase of 40 min compared to broadcast and cable television consumption. With the popularity of video streams, a new marketing method has emerged in social commerce, primarily through live-streaming sales events. A report from Sensor Tower indicates that global in-app purchase revenue from social applications reached a record USD 4.5 billion in the first quarter of 2024. This significant economic contribution highlights the importance of video streams in supporting business development within the realm of social commerce.

Social commerce represents an emerging business model that blurs the boundary between online interaction and online shopping, enabling users with a comprehensive platform to participate in social activities while simultaneously making purchases [1]. Unlike e-commerce, the defining feature of social commerce platforms is sociability, which fosters user engagement and attracts consumers. Within this context, users take active participation in various online social activities, including liking, product reviews, topic engagement, experience sharing, and service recommendations [2]. In contrast to social media, social commerce platforms offer transactional functions, directly eliminating the need for users to switch between social and shopping platforms, making “shopping while socializing” a reality. Innovative formats like short videos with purchase links and live-streaming sales have spawned a postmodern consumption model characterized by dynamic consumption behavior.

The evolution of social commerce formats has transitioned research focus from text to images and, now, to video streams. These social commerce platforms are characterized by sociability, which serves as a catalyst for attracting users and fostering interactions. Previous studies have suggested that user-generated content (UGC) on social commerce platforms can provide valuable information and online social support, helping consumers to make decisions [3,4]. It has been found that the linguistic style and emotional impact of text can attract browsing and following [5]. Consumers frequently see the benevolence and familiarity conveyed through UGC as the primary way of reducing online social uncertainty [6]. Images significantly enhance the dynamism of UGC, capturing users’ attention and engagement [7,8]. Research has shown that image features can lead to more positive attitudes towards online shopping and increased purchase intentions [9]. Video streams have introduced new impacts on social commerce by offering superior visual effects, sociality interaction, entertainment value, and immersive experience [10]. Some studies on live streaming have concentrated on the impact of streamers on consumers’ online perception and behavior. Streamer performances (such as real-time interaction and value co-creation) and streamer characteristics (such as attractiveness, authenticity, and credibility) have been found to positively affect consumers’ willingness to watch and purchase [11,12,13]. Time pressure created by limited offers and real-time interactions in live streaming has been identified as a significant factor prompting consumers to make impulsive purchasing decisions, which often result in regret due to a lack of rational consideration [11,14]. This may compel some consumers to escape from the live-streaming environment in favor of short videos. The significance of video streams in social commerce is growing, but there are only a few studies exploring the relationship between live streaming and consumers, often overlooking short video formats. Additionally, most existing studies only focus on the impact of streamer characteristics from a single perspective, ignoring consumers’ emotional and rational responses in a video stream environment. Both short videos and live streaming provide an engaging environment and immersive experience for online shopping, with the atmosphere and content features playing crucial roles in influencing consumer behavior. The process of making purchasing decisions is complex and influenced by both emotions and cognition. Therefore, in this study, we aim to analyze the mechanism of the impact of video streams on consumer purchase intention in social commerce platforms through the dual pathways of affection and rationality. This will provide a comprehensive perspective on consumers’ perception of video streams and bridge the current research gap.

This study focuses on the prevalence of short videos and live streaming within video streams and explores the impact of atmospheric and informative cues on consumers’ purchasing intention in social commerce platforms. The research draws upon Bourdieu’s Field Theory [15], providing a new approach to constructing an integrated conceptual model. Individual practices are intrinsically linked to the underlying logic of the field. In online societies, such as social commerce platforms, social interactions shape the internal structure of communities, giving rise to unique atmospheric characteristics and styles. The varying possession of social capital, such as knowledge and information, between content producers and consumers leads to differences in their online status and power [16,17]. These factors collectively influence individuals’ habitus within the field, a phenomenon explained by Field Theory [18]. Within the context of video streams, atmospheric cues evoke positive emotional responses, and dense information is processed through cognitive comprehension, which presents a complex challenge to explain from a single perspective [19]. This study concentrates on the critical elements of atmospheric characteristics and social capital, specifically investigating the impact of emotions, social presence, information quantity, and information quality. These factors directly influence the perception of similarity and power among individuals within the field through affective and rational pathways, respectively. They also indirectly shape the habitual responses to video streams, particularly consumers’ purchase intentions.

This study holds several important implications. First, we have drawn inspiration from Bourdieu’s work to develop a model that is relevant to online society. Social commerce platforms represent a form of existence within the online society, or a sub-field of the online field. We consider information as a form of capital and atmosphere as a defining characteristic of the field, which collectively influence consumers’ habits and perceptions. Second, we identified the common atmospheric characteristics of short videos and live streaming, addressing the research gap on the short video format in previous studies. We acknowledge the often-overlooked emotional expression in video streams. We explore the mechanism by which emotional contagion and social presence influence consumers’ affective responses, thereby expanding upon previous research. Last, we delve into the mechanism through which similarity and power affect user perception from affective and rational pathways. This compensates for the one-sidedness that may be brought about by single-path analysis and provides a comprehensive understanding of the online behavior of social commerce consumers.

## 2. Literature Review and Theoretical Background

### 2.1. Social Commerce

Social commerce is a new business model that involves the use of social media and Web 2.0 to support online interactions and information sharing among users, which assists in the buying and selling of products and services [20]. There are two main forms of social commerce [21]. The first form involves social networking sites such as TikTok and Xiaohongshu, which incorporate commercial functionalities and offer convenience for transactional and marketing activities for users with a social orientation. The second form involves e-commerce websites that are enhanced with social features to encourage online interaction and content contribution among consumer-oriented users, as seen in Amazon and Taobao. Unlike conventional e-commerce, social commerce emphasizes social attributes. Users can observe, comment, and interact at various stages of the purchasing process [22]. This not only enhances product sales but also attracts user engagement.

The rise of social commerce has shifted the focus to online content as a key element. Contrary to the traditional “broadcast” approach of e-commerce, social commerce relies on interpersonal penetration through two-way communication. This “shopping + interaction” mode has led to a noticeable increase in the creation of online content. Given the inherent uncertainty and risk in the digital realm, consumers see online content as a crucial resource and consult it during decision-making processes [23]. For instance, many consumers initiate their online shopping by seeking product reviews from others, presuming this information to be more trustworthy [3,23]. Consequently, online content emerges as significant stimuli influencing consumers’ psychological states. Research indicates that factors such as the volume, sentiment, and expertise of the content can differentially impact users’ perceptions and preferences [24]. Moreover, digital metrics associated with the content are instrumental. Metrics like the number of likes play a significant role in shaping a seller’s reputation and sales volume [25]. With the evolution of information technology, there has been a diversification of content forms, including text, images, short videos, live streams, and combinations of these formats. This diverse content has a significant impact on user behavior and the development of social commerce [9,14].

Research in the field of social commerce focuses on understanding the factors that influence consumers’ perceptions and behavior online. Environmental psychology suggests that people’s behaviors are shaped by their surroundings. The online environment of social commerce is different from traditional retail settings. When consumers shop online, the virtual atmosphere can evoke emotional responses, affecting their intentions and behavior [26]. For example, the sense of social presence on a social commerce platform can simulate the feeling of being physically present with other consumers in a retail environment, leading to impulsive buying [27]. In addition, social commerce constructs, perceived value, and online interpersonal influence significantly contribute to enhancing perceived familiarity, fostering online social interaction, and promoting purchases [28,29]. These studies demonstrate that online environmental cues play a significant role in shaping consumer perceptions of value, which, in turn, influence their buying decisions. Even in newer forms of social commerce like video streams, the subjective perceptions elicited by online environment and interaction continue to impact consumers’ purchase intention [14,30].

### 2.2. Field Theory

Bourdieu’s Field Theory provides a comprehensive framework for examining social movements, incorporating important concepts such as field, capital, and habitus. In Bourdieu’s language, the field represents “a set of objective, historical relations between positions” [31]. It is perceived not merely as a structure but also as an environment where different individuals, groups, and institutions coexist and mutually influence each other. Consequently, the field possesses unique characteristics. Capital within this field goes beyond mere resources and economic wealth. It encompasses social networks that consist of “more or less institutionalized relationships of mutual acquaintance and recognition” [31,32]. An individual’s specific position in the field is closely tied to the distribution and acquisition of capital [16]. Individuals with more resources and capital tend to occupy more central position, and their capital and position also shape an individual’s perceptual framework and behavioral tendencies, as defined by habitus. Habitus is “an endless capacity to engender products-thoughts-perceptions-expressions-actions”, which is employed to link the “objective” and the “subjective” social realms [33]. Individuals in this field hold diverse positions influenced by their possession of capital, which, in turn, modulates their internalization processes and ultimately shapes their behaviors. Generally, individuals in the field aspire to accumulate more capital as it can help to occupy a more favorable position in social networks. Whether it is the inherent appeal of capital or an individual’s intrinsic desire for it, capital subtly shapes their internal thoughts and even actual behaviors. In essence, Field Theory underscores the subtle transformative effect of capital on individuals’ habitus.

In the evolving digital society, researchers increasingly utilize Bourdieu’s theory as a foundational framework for their studies. Levina and Arriaga (2014) introduced the concept of the online field, analyzing the production process of social status within UGC platforms from this perspective [17]. They explained the relationship and distinction between status and power, stating that power engenders status, while status, in turn, generates power. Visible indicators of differentiation serve as markers of status, such as garnered attention on social media platforms, with the status being a manifestation of power. Furthermore, the study of digital society also incorporated the concept of capital. van Dijk (2006) proposed a comprehensive definition of information capital, encompassing both financial capital and intangible capital [34]. Unlike financial capital used to cover computer and network costs, intangible resources have more abstract characteristics, including technical skills, evaluation ability, and implementation capability. While intangible capital differs from economic or cultural capital, it can be transformed under certain conditions [35]. For instance, content producers garner network attention through social media activities, and the attention economy turns it into monetary gain through advertising revenue, or other means. The concept of habitus is equally crucial in digital society research, facilitating our understanding of how individuals utilize Internet resources. This encompasses the examination of how the information habitus of individuals in different locations in social networks is formed, such as the habitus of information seeking and online career planning [35,36]. When individuals form internalized perceptions in a digital society, the concept of digital habitus becomes indispensable for predicting future actions, decisions, and directions [35].

Field Theory emphasizes that interactions between individuals are influenced by various social structures and conditions. Arnould and Thompson (2005) explained how social structures impact consumption [18]. They suggested that social class shapes people’s values and influences their choices as consumers. According to Bourdieu, online practices are inseparable from the underlying logic of all human action domains. Online platforms, such as social media, predominantly comprise two groups: content producers and content consumers. The social interactions between these two groups are a defining characteristic of online communities. Content producers establish their own “soapboxes” by posting content, giving them a higher status and more attention compared to content consumers [37]. The content they produce serves as a form of social capital, whether knowledge or personal experience. Content consumers, on the other hand, contribute to the producers’ social capital through interactions like liking and commenting. They also increase their knowledge capital by integrating content into their cognitive systems through tagging. Overall, in Bourdieu’s framework, these “commodities” associated with symbolic wealth and position are considered social capital. And the phenomenon of “liking” mirrors the recognition structure within social commerce platforms [38,39,40]. The generation of “likes” is a collaborative endeavor involving individuals associated with the field, each contributing to the accumulation of these social approvals.

Building upon the aforementioned studies, this study aims to develop a comprehensive framework that explains how the online atmosphere and information influence consumer purchase intentions in social commerce, specifically in the context of video streaming. To achieve this, we conceptualize the social commerce platform as a unique field with a distinct atmosphere and style, drawing upon Bourdieu’s perspective. Online information and the associated “likes”, “comments”, “danmu” and “favorites” serve as forms of social capital. When consumers engage with the field, the atmosphere and social capital will influence their perceptions both emotionally and rationally, potentially changing their beliefs and behaviors (i.e., habitus). In our study, we investigate consumer purchase intention in social commerce platforms, with similarity and power serving as mediating factors.

## 3. Hypotheses Development

In the following sections, we construct hypotheses from three key aspects to explore the relationship between atmosphere characteristics, social capital, and individual habitus. In terms of atmosphere characteristics, emotion and social presence are selected as representative variables. We analyze both the quantity and quality of social capital in social commerce separately to obtain a comprehensive understanding. For habitus, we consider the perception of similarity and power as mediating variables. Purchase intention is the dependent variable in this study.

It is important to highlight that similarity usually pertains to the homophily of demographic or psychological traits, such as age, gender, lifestyle, or personality [41]. But, in this study, we concentrate on internal similarity, which refers to the subjective depiction of an individual’s intrinsic characteristics, like attitude, values, interests, and preferences [42]. While watching video streams, consumers may unconsciously compare themselves to the streamers in the visual content. For example, if a product recommended by a streamer in the video matches the consumer’s personal color preferences, they might perceive a similarity in their color choices to that of the streamer. This study aims to measure the degree of similarity between consumers and streamers. On the other hand, it is challenging to directly represent an individual’s power, economic status, or social class within networks. Individuals always derive guidance by observing the behavior of influential social media users. Consequently, we interpret power in online communities as the capacity to influence others [43]. Typically, streamers have a deeper knowledge and experience with products compared to consumers, and they also possess a strong appeal that can drive purchases. Consumers may be influenced by the streamer and make purchasing decisions based on this influence. Therefore, power is defined as the consumer’s perception of the streamer’s influence in this study.

### 3.1. Atmosphere Characteristics and Similarity

Emotion refers to mental states with strong activation and intrinsic drive that fully engage in activities that are meaningful for individuals [44]. Beyond facial expressions, emotions can also be expressed and transmitted through a variety of media, such as gestures, words, pictures, and music [44,45]. Emotion is essentially a response to the perception of an individual. The contagiousness of it allows individuals to experience emotions similar to those of the source, although they may not immediately realize it. From a relational perspective, emotions are regarded as “relational scenarios”, indicating that they are inherently collective [46]. Collectively dealing with emotions is increasingly prevalent in digital media. Emotion serves as a socio-historical performance, intricately intertwined with relations and contexts to form an atmosphere [46,47]. Consequently, we consider emotion as a representative variable for atmosphere cues in video streams.

Emotion plays a crucial role in social media and online website research. It is a significant element in attracting audiences and fostering engagement [48]. Barsade (2002) emphasized that the expression of excitement, enthusiasm, and passion can influence opinions and subscriber numbers due to the phenomenon of emotional contagion [49]. Emotions conveyed through digital content not only provide valuable cues for readers’ cognitive processing but also facilitate the transmission of emotions from earlier users to subsequent ones [50]. When individuals are requested to post comments following prior feedback, their subsequent written responses often show biases influenced by the emotional similarity observed [51]. Emotional contagion leads to empathy by triggering psychological reactions, which enables individuals to understand and align with others’ emotions [52]. Therefore, we argue that the presence of positive emotions in video streams on social commerce platforms triggers this contagion, prompting users to adopt similar emotions, perspectives, and attitudes. Building on this premise, we propose the following:

**H1.**  *Emotion can increase similarity in social commerce.*

Social presence means the extent to which users perceive the psychological presence of others through a medium [53]. According to social presence theory, factors like intimacy and immediacy influence perceived warmth on social media platforms. Social media platforms include various features, such as liking, commenting, and sharing, to enhance social presence among users. In this study, we specifically examine the consumer’s perception of social presence when using social commerce platforms. In this digital context, social cues can engender a virtual social presence, which is perceived as an integral component of the online atmosphere [54]. The intensity of this social presence may change depending on the setting [55], so we consider social presence as another representative variable of atmosphere cues in video streams.

Social commerce platforms, while not involving direct interaction with other individuals, still have the potential to create a sense of social presence. It is often observed that interactions between individuals create a stronger sense of presence compared to interactions facilitated by user–medium communication. In addition, the frequency of interpersonal interactions in online environments correlates directly with users’ perceptions [28,56]. User comments and contents on social commerce platforms, as forms of interpersonal interaction, have been demonstrated to have a causal relationship with social presence. An increase in the number of customer reviews within an online concert ticket-sales environment was found to make customers perceive social presence [28,57]. Particularly in the form of short videos and live streaming, the role of social presence is more prominent [58]. Social commerce platforms provide opportunities for content producers to self-disclose and share experiences. Consequently, they can utilize interactive language to establish personal connections with other users and generate a perceptible social presence. This identifiable cue creates a sense of similarity towards content producers in consumers’ minds [58]. Therefore, we propose the following:

**H2.**  *Social presence can increase similarity in social commerce.*

### 3.2. Social Capital and Power

Social capital, as defined by Coleman, refers to the connections among users and resources within a social network that bring value or benefit [38,59]. Unlike monetary value in economics, social capital is more accurately understood as the accumulation of mutually supportive behaviors and norms among individuals within a group. This study will evaluate social capital from two perspectives: quantity and quality.

The quantification of social capital includes elements such as the amount of information and the count of likes, comments, favorites, etc. Numerical metrics have a significant influence, and conformity readily occurs on social media. According to social impact theory, the combined impact of social power on individuals within a given domain is frequently contingent upon the quantity, potency, and directness of others involved. The centrality ranking of an individual aligns asymptotically with its social power ranking, indicating that those with greater social capital in digital society will demonstrate higher levels of power [60]. When an individual possesses valuable information and receives recognition for it, they assume the role of opinion leader and produce a capacity to affect the attitude and behavior of others [61,62]. For example, the number of followers an influencer has can influence the intentions and actions of online consumers, including product engagement, purchase decisions, and dissemination of eWOM [63]. Furthermore, more introduction word counts, likes, and online reviews in crowdfunding scenarios can enhance public trust in the project [64]. Drawing from these observations, we propose the following hypothesis:

**H3.**  *The quantity of social capital can increase power in social commerce.*

We employ the concept of information quality to represent the quality of social capital, specifically focusing on consumers’ perceptions of reliable information. This typically encompasses factors, such as relevance, comprehensibility, sufficiency, and objectivity [65]. Online information, as a form of digital social capital, offers benefits including emotional support, diverse perspectives, and non-redundant information [66]. Despite variations in actual information quality, tweets and comments have emerged as crucial references for individuals, particularly before making purchasing decisions. The positive influence of quality signals from online information and WOM is substantial and directly impacts the perception of reliability and trustworthiness [64,67]. Generally, higher-quality information is more persuasive to its recipient. Therefore, it can be inferred that the quality of content in video streams can influence and shape consumers’ perceptions of power. Based on these findings, we propose the following:

**H4.**  *The quality of social capital can increase power in social commerce.*

### 3.3. Similarity and Purchase Intention

In the field of social psychology, it is widely accepted that individuals perceive an event as psychologically closer when it happens to someone similar to them. This highlights the importance of similarity in human perception. For example, in traditional retail settings, the similarity between a salesperson and a consumer can significantly increase the persuasiveness of suggestions [68]. Given that the beneficial impact of external similarity may diminish or even be reversed, our study emphasizes the internal similarity among social media users [69]. The interpersonal interaction among members of digital communities is crucial. Similarity, as a factor that attracts people to one another, it fosters intangible emotional connections while strengthening perceptions of intimacy or bond strength. Therefore, the perception of similarity can help counter the uncertainty of information sources in network environments [70]. Furthermore, similarity has positive effects on the usefulness and trust perceived by online users. For instance, in online movie ticket sales, similarity can effectively increase consumers’ intention to make a purchase [71]. Therefore, we argue that similarity enhances consumers’ acceptance of recommended products or services in social commerce platforms. We propose the following:

**H5.**  *Similarity can increase purchase intention in social commerce.*

### 3.4. Power and Purchase Intention

On social media platforms, ordinary users typically possess less power compared to influential users who garner a significant number of “likes” and “comments”. Individuals with lower power often seek to become more similar to those with higher power, thereby increasing their likelihood of assimilation when comparing various characteristics [72]. For instance, consumers with a low-power status tend to bridge the gap between high-power-status individuals through compensatory consumption. Studies have shown that power plays a positive role in the relationship between status consumption and the intention to make a purchase. Specifically, power serves as a reliable indicator that satisfies users’ cognitive needs for online information and trust when confronted with an abundance of information on the network [38,73]. As a result, social media users are more likely to accept such recommendations or be persuaded by identifying the expert power that is conveyed in the online information.

**H6.**  *Power can increase purchase intention in social commerce.*

### 3.5. Research Model

**Figure 1:**
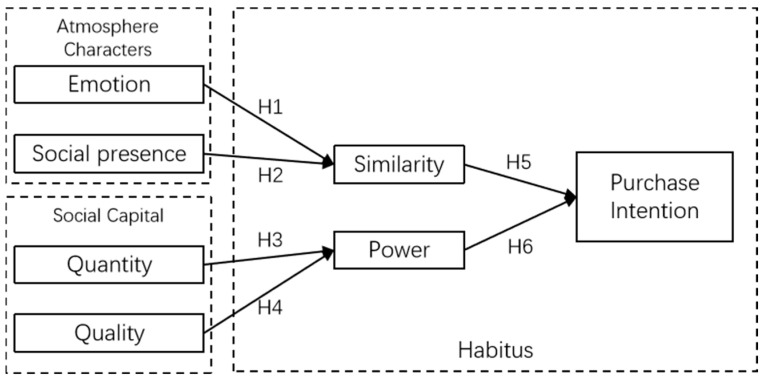
Research model.

Based on Field Theory and the above hypotheses, we construct the conceptual model. Figure 1 illustrates the relationships among constructs.

Consumer purchase intention is shaped by both affective and rational factors. Affectively, video streams can create a sense of social connection among consumers and potentially enhances their positive emotional experiences [19]. These positive emotions can lead to an increased sense of closeness and perceived similarities among consumers, which can, in turn, increase the likelihood of them making a purchase. Positive affective responses can speed up the decision-making process and enhance enjoyment and satisfaction [74]. On the rational side, the video-streaming environment is information-intensive, and consumers need to use their cognitive abilities to process this information effectively. The perceived quality of the information can positively impact consumers’ cognitive reactions [19]. Moreover, the volume and quality of the information determine its persuasiveness, and highly persuasive content can be more powerful in driving consumer purchase intentions. Consequently, we hypothesize that similarity and power play mediating roles in the affective and rational paths, respectively.

## 4. Methodology

This study aims to explore the subjective perceptions of consumers in social commerce platforms, which cannot be directly observed. Consequently, we posit that a questionnaire survey is the most appropriate method for data collection.

### 4.1. Research Design

Our survey consists of two phases. In the first phase, we asked participants to recall their most recent shopping experience on a social commerce platform through short videos or live streaming. Subsequently, participants answered verification questions to ensure that they had noted serious recollections.

In the second phase, we asked participants to complete a questionnaire. The items of the main body of the questionnaire were scored on a 7-point Likert scale and adapted from well-established scales. The items were contextualized and adapted according to expert advice, to be able to better fit the context of this study. The details of the questionnaire are reported in Table 1. We also investigated questions about personal information and Internet user experience. These questions covered demographic factors such as gender, age, and educational background, as well as the frequency of social commerce platform usage.

### 4.2. Participants and Data Collection

The participants in this study were users of social commerce platforms in China, such as Xiaohongshu, Douyin, Kuaishou, etc. We followed a rigorous translation process and collaborated with language experts to ensure accuracy. The questionnaire was conducted in two stages. Initially, a pretest was carried out to select 60 users for the pilot survey. The reliability and validity of the questionnaire were verified through the pretest results. Subsequently, the final questionnaire was officially launched. We employed Credamo (www.credamo.com), a professional online survey platform in China, to design an electronic questionnaire. Cash incentives were provided to participants who completed the survey.

Several measures were taken to ensure the quality of this survey, including:

1. Prohibition of repeated participation;
2. Restriction of credit scores to ensure only eligible individuals could participate;
3. Integration of validation questions to eliminate careless or irrelevant responses;
4. Establishment of human–computer verification mechanisms to deter automatic generation or other forms of computer-generated responses;
5. Request for participants to complete all items within the questionnaire.

Non-compliance with these stipulations would lead to immediate suspension and termination of the survey.

We conducted two rounds of survey research. The first round was executed from 15 March to 22 April 2023, and the second round was carried out from 6 June to 20 June 2023. Throughout this period, we collected a total of 623 questionnaires. A screening process was meticulously conducted on these questionnaires based on the following criteria:

1. Failure to pass the validation question;
2. Answer time less than 360 s or more than 900 s;
3. More than 80% of the questions were assigned an identical value.

Following the screening process, we obtained a total of 515 valid questionnaires, resulting in a response rate of 82.66%. Table 2 provides a summary of the sociodemographic characteristics of the sample. The sample is predominantly female and primarily young in age. The majority of participants hold a bachelor’s degree. More than half of the participants reported using social commerce platforms at least once per day.

To confirm the validity of this study, we initially utilized Harman’s single-factor test to assess the existence of common method bias [77]. The unrotated factor solution revealed the existence of multiple factors, with the first factor accounting for only 29.23% of the total variance, below the threshold of 50%. We further investigated this issue with a structural equation model (SEM) analysis, which had a low fit (χ^2^/DF = 9.98; RMSEA = 0.132; NFI = 0.496; CFI = 0.520; IFI = 0.522). Consequently, based on these two examination methods, we concluded that any common methodological bias was insignificant.

## 5. Data Analysis

### 5.1. Validation of the Measuring Scales

Initially, we conducted an examination of the reliability and validity of the scale utilizing SPSS 27.0 and AMOS 26.0 software. The results are recorded in Table 3. The Cronbach’s alpha (α) coefficient values for each variable surpassed the recommended threshold of 0.7, validating the reliability of the scale. This reliability was further corroborated by confirmatory factor analysis (CFA). Both the composite reliability (CR) coefficient and the average variance extracted (AVE) exceeded the recommended threshold of 0.7 and 0.5, respectively, suggesting a high degree of internal consistency.

In terms of validity, each item demonstrated significant results (*p* < 0.01), with standardized loadings exceeding 0.70, thereby demonstrating robust convergent validity (Table 3). Furthermore, we corroborated discriminant validity through a comparison of the arithmetic square root of the AVE values and correlation coefficients, as reported in Table 4.

In conclusion, the results offer compelling evidence for the reliability, convergent validity, and discriminant validity of the measures employed in this study.

### 5.2. Hypothesis Test

The conceptual research model was analyzed using SEM, and the results indicated that the data fit well with the conceptual model: χ^2^/DF = 2.165; RMSEA = 0.048; GFI = 0.919; IFI = 0.939; TLI = 0.930; CFI = 0.919. We used AMOS to analyze the hypothetical model. The results are recorded in Table 5.

In the context of atmosphere characteristics, the standardized path coefficient between emotion and similarity is denoted as β = 0.165 (*p* < 0.01), thereby signifying a significant positive correlation between them. Therefore, H1 is supported. Moreover, the findings reveal that social presence exerts a substantial and positive influence on similarity (β = 0.411, *p* < 0.001), which is consistent with H2. Thus, it can be inferred that atmosphere cues in the social commerce field, such as emotion and social presence, have the potential to enhance consumers’ perception of similarity.

In terms of social capital, the standardized path coefficient β = 0.330 (*p* < 0.001) for the quantity of capital on power suggests a significant relationship. This implies that the volume of online content, as measured by engagement (such as likes, favorites, comments, and danmu), significantly enhances power, supporting H3. The coefficient effect of the quality of online content on power is also significant and positive (β = 0.240, *p* < 0.001). This finding proves the positive influence of online content quality on power, thereby supporting H4. These observations imply that both the quantity and quality of capital in social commerce can enhance consumers’ perception of power.

Regarding purchasing intention, the findings revealed that users’ perceived similarity positively influenced their purchase intention (β = 0.328, *p* < 0.001), thereby supporting H5. The standardized path coefficient between power and purchase intention was found to be β = 0.310 (*p* < 0.001), indicating that power can facilitate users in accepting the recommendation of a product. This result supported H6. In conclusion, both similarity and power can enhance consumers’ purchase intention on social commerce platforms.

### 5.3. Mediating Effect Test

Given the pivotal role of similarity and power in bridging the relationship between atmosphere characteristics, social capital, and user intention within the model, we undertook further analysis to elucidate their mediating effect. Utilizing bootstrapping estimation with 5000 large samples in SPSS, we separately examined the existence of similarity and power mediation effects in affective and rational paths. We present the mediating effect across four paths, with Table 6 documenting the robust estimation results.

The findings suggest that the mediating effect of similarity in emotion’s impact on purchase intention is significant, as 0 is not encompassed within the confidence interval. Similarity partially mediates this relationship, accounting for a 32.31% proportion of the total effect. Similarity also partially mediates the relationship between social presence and purchase intention, and the proportion of the mediating effect is 41.47%. In terms of social capital, the impact of quantity on purchase intention is partially mediated by power, and its effect proportion is 30.08%. In the impact of quality on purchase intention, the mediating effect of power is also significant. Power plays a partial mediating role, and the proportion is 21.01%.

## 6. Discussion

This observation of atmospheric characters aligns with previous research emphasizing the significance of emotional cues in social media platforms [55,57,61,78]. Social commerce is characterized by its emphasis on social interaction and two-way communication. In video-streaming contexts, streamers and content creators may intentionally or unintentionally infuse their emotions into content and expression, which can be perceived by observers. Video streams with high vividness and interactivity create a strong sense of presence, thereby influencing consumers’ affective responses. This, in turn, enhances consumers’ perception of similarity and encourages them to participate and make purchases. Such positive interactions contribute to a harmonious online environment, potentially influencing external observers in similar ways. This perspective is consistent with Bourdieu’s theory, which suggests that the harmonious online atmosphere resulting from social interactions gives social commerce platforms distinct characteristics and subtly influences consumer habitus.

We recognize that online content plays a significant role as a form of social capital in digital society, especially on social commerce platforms. This finding supports the idea that power has a positive influence [38,72,73]. In a network where uncertainty is common, users tend to see high-quality and endorsed information as more authoritative [79]. The inherent persuasive power of high-quality recommendation information is further strengthened by recognition, such as likes. The recognition not only increases the volume of capital but also enhances users’ perception of power. Our study provides strong evidence for the positive effects of power rather than its negative impacts. These findings also further support the applicability of Bourdieu’s theory that the influence of social capital on individual habitus remains intact in the digital society.

In social commerce platforms, we associate similarity with consumers’ affective responses and power with their cognitive evaluations. In the affective pathway, the effects of emotions and social presence in video streams on consumers’ behavioral intentions are partially mediated by similarity. On the other hand, power plays a partial mediating role in the rational pathway. Social commerce consumers are influenced not only by the content in video streams but also by the implied power, both of which significantly influence their subsequent decision making. This study broadens the scope of previous research by compensating for the limitations of a single perspective [10,12,13]. Examining different affective and rational pathways offers a more detailed understanding of the processes influencing consumers’ perceptions and decisions in social commerce.

## 7. Conclusions

This research explores the factors influencing consumers’ intention to purchase while watching video streams, utilizing Bourdieu’s Field Theory as the theoretical foundation. This study emphasizes the role of field atmosphere and social capital in shaping consumer habitus through affective and rational pathways. A survey of 515 social commerce consumers led to the following conclusions:

First, the emotion and social presence within field atmosphere positively impact the perception of similarity. And the increase in similarity enhances consumers’ purchase intention in video streams. Second, our research shows that the quality and quantity of video content on social commerce platforms positively influence the perception of power. This perception of power increases the likelihood of consumers intending to buy the recommended product. In addition, we examine the partial mediating role of similarity and power in the process of accepting stimuli, forming perceptions, and making decisions. Individuals typically process information through both affective and rational pathways.

These findings provide insights into the heterogeneity of video streams’ impact on individuals and help to explain why people can have different reactions to the same information. For example, when multiple individuals come across the same recommended content on social commerce platforms, some may be likely to follow the recommendations while others may ignore them. These varying responses can be attributed to the unique psychological perception of the receiver of an information source, which encompasses both similarity and power. 

## 8. Implications

### 8.1. Theoretical Implications

Social commerce plays an increasingly important role in shaping consumers’ purchase intentions and behaviors. Our study makes significant theoretical contributions to this field. Given the multiple factors present in video streams of social commerce platforms, we have introduced a comprehensive research model. This model incorporates both affective and rational elements to address the limitations of a singular perspective in the prior literature. We explain the process of how consumers’ behavioral intentions, especially purchase intentions, are formed through atmospheric cues and social capital. We separately validated the positive impacts of emotion, social presence, and information quantity and quality within affective and rational pathways. Our findings offer valuable insights and a comprehensive understanding of consumer behavior in social commerce platforms.

This study contributes to the development of Field Theory in the context of social commerce. We present a theoretical framework based on Bourdieu’s Field Theory to explain the outcomes of interpersonal interaction and the subsequent influence on individual behavior. In recent years, scholars have expanded the application of Field Theory within the domain of information systems [17,35]. We further extend this perspective by considering social commerce platforms as a distinct field. We expand the understanding of capital and habitus within online societies and provide a complement to the digital development of Field Theory. The interaction between online content producers and consumers not only determines the structural characteristics of the field but also accumulates social capital, which, in turn, shapes individuals’ unique positions and power. From this viewpoint, we explore the influential role of the social commerce field on individual habitus, as reflected in consumer behavioral intention. Our findings confirm that Bourdieu’s theory remains relevant to the context of social commerce. This contributes to existing literature and provides a new perspective for understanding consumer behavior in social commerce.

This study investigates the impact of atmospheric cues and social capital on consumers’ purchase intention. We aim to elucidate the generation process of behavioral intention in a context where direct interaction may be absent but still exert influence. The findings indicate that similarity and power effectively play important roles in affecting consumers’ affective and rational responses, respectively. Similarity partially mediates the effects of emotion and social presence on purchase intention, while power partially explains how information influences it. This study reveals the influence of video streams in shaping consumer purchase intentions, enriching the research content within the realm of social commerce.

### 8.2. Practical Implications

Our study holds significant relevance for the advancement of social commerce. We advocate that video content creators and streamers should ground their creations in genuine emotions and personal experiences. They could try to evoke positive affective responses from consumers by incorporating emotional language into conversations. Concurrently, they should ensure content quality to maintain professionalism and credibility. This approach fosters a positive cycle: emotional stimulation and high-quality content attract consumer interaction, leading to positive feedback through digital engagement. Such feedback encourages more consumers to get involved, while creators and streamers are motivated to continue producing high-quality content due to the recognition they receive. This process fosters empathy and intimacy among consumers, amplifies purchase intent through powerful content, and ultimately facilitates sales promotion.

Brands and marketers should strategically select cooperators and employ diverse marketing strategies. According to our findings, managers should give priority to cooperating with streamers or video creators who possess superior content creation and emotional expression skills. Such partnerships can effectively attract consumers and promote product sales. In addition to live streaming, brands and marketers should also emphasize the promotion of short videos. Alternatively, they could edit the recorded live streaming into multiple short videos for various products, allowing for playback and push. This approach aims to reach potential consumers who might not be able to watch the live stream due to time pressure or other reasons.

Managers of social commerce platforms should implement reward systems and regulations. Our findings indicate that high-quality information, which receives many likes and positive feedback, significantly influences users’ likelihood of making a purchase. Consequently, the platform should prioritize high-quality content by increasing its visibility and exposure to ensure appropriate recognition. It is important to reward the creators and promoters of such content to maintain their enthusiasm and activity. In addition, regulations should be established to prevent false advertising and fraud, thereby creating a trustworthy online shopping environment for consumers.

## 9. Limitations and Future Lines

Despite the valuable insights our study provided for the research of social commerce, some limitations need to be addressed in future research. First, we need to recognize that our study relied primarily on self-report measures, which are inherently subjective and susceptible to various biases. The selected scales were contextually adapted to fit the research background, especially the emotion scale. We hope that future research will develop more appropriate scales for measuring emotions in video streams. Additionally, we did not include objective data (e.g., actual purchasing behavior) in our analysis. This suggests that future studies could integrate objective measurements or observational data with self-reports for a more holistic understanding of user behavioral patterns in digital society.

Second, we only investigated one type of social commerce in our survey: social websites with business functions. However, users may have different perceptions and decision-making processes when utilizing other types of social commerce platforms such as Taobao. Consequently, future research should encompass a broader range of social commerce platforms to uncover more universally applicable rules.

Finally, our study primarily concentrates on the impact of recommendation information while leaving the influence of negative information to future exploration. Contrary to positive content, negative information triggers different affective responses and rational evaluations. These differences could potentially influence users’ online perceptions and subsequently affect their behavioral intentions. This will also serve as an important direction for future research.

## Figures and Tables

**Table 1:** Variable measurement.

Constructs	Items	Measurement	Reference
Emotion	EMO1	I feel the content in short video/live streaming touch me.	Lee and Theokary [5];
	EMO2	I feel the content in short video/live streaming move my emotions.	
	EMO3	I can feel the emotion from content in short video/live streaming.	
Social presence	SP1	I feel a sense of human contact in short video/live streaming.	Gefen and Straub [55]
	SP2	I feel a sense of sociability in short video/live streaming.	
	SP3	I feel a sense of human warmth in short video/live streaming.	
	SP4	I feel a sense of human sensitivity in short video/live streaming.	
Quantity	QUAN1	The number of likes of the short video/live streaming is large.	Park et al. [65]; Filieri [66]
	QUAN2	The number of favorites/focus of the short video/live streaming is large.	
	QUAN3	The number of reviews/danmu of the short video/live streaming is large.	
	QUAN4	The amount of information in short video/live streaming is large.	
Quality	QUAL1	The short video/live streaming provides timely information.	Park et al. [65]; Filieri [66]
	QUAL2	The short video/live streaming provides accurate information.	
	QUAL3	The short video/live streaming provides useful information.	
	QUAL4	The short video/live streaming provides relevant information.	
Similarity	SIM1	As for styles about the products/services, I feel similar with the streamer in short video/live streaming.	Hu et al. [71]
	SIM2	As for tastes about the products/services, I feel similar with the streamer in short video/live streaming.	
	SIM3	As for likes and dislikes about the products/services, I feel similar with the streamer in short video/live streaming.	
	SIM4	As for preferences about the products/services, I feel similar with the streamer in short video/live streaming.	
Power	POW1	I think the streamer in short video/live streaming knows more about the products/services than I do.	Raven et al. [75]
	POW2	I think the streamer in short video/live streaming has more expert knowledge of the products/services than I do.	
	POW3	I think the streamer in short video/live streaming has more information and experience about the products/services than I do.	
Purchase Intention	PI1	I intend to purchase the products/services in short video/live streaming.	Pavlou and Fygenson [76]
	PI2	I plan to purchase the products/services in short video/live streaming.	
	PI3	I predict that I would purchase the products/services in short video/live streaming.	
	PI4	It is highly likely I would purchase the products/services in short video/live streaming.	

**Table 2:** Analysis of demographic variables.

Variable	Category	Absolute	Percent (%)
Gender	male	191	37.09
	female	324	62.91
Age	<20	24	4.67
	20–29	233	45.24
	30–39	206	40.00
	40–49	39	7.57
	≥50	13	2.52
Education background	Primary and below	3	0.58
	Junior High School	10	1.94
	Senior High School	60	11.65
	Undergraduate degree	351	68.16
	Master’s degree and above	91	17.67
Times of weekly use	once a week or less	19	3.69
	2–3 times a week	79	15.34
	4–6 times a week	140	27.18
	once a day or more	277	53.79

**Table 3:** Confirmatory factor analysis.

Factor	Indicator	Loading	α	AVE	CR
Emotion	EMO1	0.755	0.803	0.583	0.807
	EMO2	0.742			
	EMO3	0.792			
Social presence	SP1	0.709	0.822	0.536	0.822
	SP2	0.720			
	SP3	0.735			
	SP4	0.763			
Quantity	QUAN1	0.752	0.843	0.576	0.845
	QUAN2	0.775			
	QUAN3	0.759			
	QUAN4	0.750			
Quality	QUAL1	0.721	0.811	0.518	0.811
	QUAL2	0.732			
	QUAL3	0.715			
	QUAL4	0.710			
Similarity	SIM1	0.762	0.838	0.565	0.838
	SIM2	0.735			
	SIM3	0.763			
	SIM4	0.746			
Power	POW1	0.776	0.814	0.592	0.813
	POW2	0.764			
	POW3	0.769			
Purchase intention	PI1	0.723	0.835	0.561	0.836
	PI2	0.755			
	PI3	0.753			
	PI4	0.764			

**Table 4:** Descriptive statistics and correlations.

	EMO	SP	QUAN	QUAL	SIM	POW	PI
EMO	**0.763**						
SP	0.398	**0.732**					
QUAN	0.372	0.590	**0.759**				
QUAL	0.316	0.333	0.395	**0.720**			
SIM	0.295	0.446	0.330	0.672	**0.752**		
POW	0.215	0.494	0.388	0.350	0.447	**0.770**	
PI	0.300	0.328	0.350	0.431	0.433	0.414	**0.749**

Note: The bold values on the diagonal are the arithmetic square roots of AVE. Off-diagonal elements are the correlation between the respective constructs.

**Table 5:** Path analysis of basic model.

Hypothesis	Path	Standard Coefficient	S.E.	C.R.	*p*	Result
H1	Emotion → Similarity	0.165	0.061	2.892	**	Supported
H2	Social presence → Similarity	0.411	0.064	6.740	***	Supported
H3	Quantity → Power	0.330	0.060	5.708	***	Supported
H4	Quality → Power	0.240	0.077	4.104	***	Supported
H5	Similarity → Purchase intention	0.328	0.054	5.797	***	Supported
H6	Power → Purchase intention	0.310	0.046	5.487	***	Supported

Note: ** *p* < 0.01; *** *p* < 0.001.

**Table 6:** Mediation results.

Path	Path A	Path B	Path C	Indirect Effect				
	X → M	M → Y	X → Y	95% Confidence Interval				
	Coeff	Coeff	Coeff	Effect	S.E.	Lower	Upper	Rate
Emotion → Similarity → Purchase intention	0.248 ***	0.323 ***	0.168 ***	0.084	0.028	0.038	0.146	32.31%
Social presence → Similarity → Purchase intention	0.368 ***	0.306 ***	0.157 ***	0.104	0.031	0.052	0.171	41.71%
Quantity → Power → Purchase intention	0.321 ***	0.279 ***	0.208 ***	0.084	0.024	0.042	0.134	30.08%
Quality → Power → Purchase intention	0.283 ***	0.265 ***	0.282 ***	0.082	0.026	0.037	0.142	21.01%

Note: 5000 bootstrap samples with 95% confidence interval. *** *p* < 0.001. Rate means the proportion of indirect effects in total effects.

## Data Availability

The raw data supporting the conclusions of this article will be made available by the authors on request.
